# Patellar malalignment correlates with increased pain and increased synovial stress hormone levels–A cross-sectional study

**DOI:** 10.1371/journal.pone.0289298

**Published:** 2023-07-27

**Authors:** Marco Brenneis, Marius Junker, Rebecca Sohn, Sebastian Braun, Markus Ehnert, Frank Zaucke, Zsuzsa Jenei-Lanzl, Andrea Meurer

**Affiliations:** 1 Department of Orthopedics (Friedrichsheim), University Hospital Frankfurt, Goethe University, Frankfurt/Main, Germany; 2 Department of Orthopedics, Tabea Hospital Hamburg, Hamburg, Germany; 3 Department of Orthopedics (Friedrichsheim), Dr. Rolf M. Schwiete Research Unit for Osteoarthritis, University Hospital Frankfurt, Goethe University, Frankfurt/Main, Germany; 4 Medical Park St. Hubertus Klinik, Bad Wiessee, Germany; University of Liege: Universite de Liege, BELGIUM

## Abstract

**Purpose:**

Risk factors for the development of pain in the context of knee osteoarthritis (KOA) remain unclear. Radiological findings often do not correlate with clinical findings, so other pathomechanisms in the development and perception of pain must play a role. The purpose of this study is to investigate the correlation of increased sympathetic nervous system (SNS) activity (measured by subjective and objective chronic stress parameters) with KOA severity, patellofemoral malalignment, and pain.

**Methods:**

47 patients with KOA were assessed. Radiological measurements of tibiofemoral and patellofemoral parameters (Kellgren-Lawrence-score, patellar tilt (PT), Caton-Deschamps-Index and Hepp´s classification) were performed and correlated with knee-specific questionnaires (WOMAC^®^, KSS^©^) and chronic stress questionnaires (PSQ-20). Additionally, parameters associated with chronic stress were quantified in synovial fluid and serum samples from patients.

**Results:**

PT correlated significantly with Caton-Deschamps-Index (r = 0.394,p = 0.006) and with medial patellofemoral joint space (r = 0.516,p<0.001). In addition, asymmetric trochlear groove (Hepp’s classification > II) was associated with significantly higher PT values (p = 0.014). A negative correlation between PT and KSS^©^-symptoms subgroup was found (r = -0.340,p = 0.024). Patients with PT<5° had significantly higher scores in the Knee Society Score^©^-symptoms subgroup (p = 0.038). A positive and significant correlation between synovial aldosterone levels and PT was observed (r = 0.548,p = 0.042).

**Conclusion:**

The results of this study indicate that patellar malalignment might correlate with increased pain. The previous specification of standard PT values must be reconsidered as even low PT values seem to play a role in the occurrence of patellofemoral osteoarthritis symptoms. Lower PT values might lead to aggravated symptoms in patients with KOA due to a narrow medial patellofemoral joint space. In addition, PT might induce the release of synovial stress biomarkers and thus contribute to the progression of KOA.

## Introduction

Osteoarthritis (OA) represents the leading chronic degenerative joint disease and is characterized by progressive loss of articular cartilage, low-grade synovitis and subchondral bone remodeling [1]. Surprisingly, the relationship between pain perception and radiologic tibiofemoral osteoarthritis (TFOA) grade in the knee is ambiguous [2]. On the one hand, the proportion of radiographic OA diagnosis in patients with knee pain ranges between 15% and 76% [3] and even 31.2% of the patients with TFOA grade IV according to Kellgren-Lawrence did not show any knee specific symptoms such as aching or stiffness [4]. On the other hand, Duncan et al. pointed out that all pain items of the WOMAC^®^ scale are associated with radiological signs of knee osteoarthritis (KOA) [5]. Thus, the risk factors for the occurrence of pain in the course of KOA remain unclear.

Patellofemoral osteoarthritis (PFOA) is a major cause of anterior knee pain (AKP) [6]. A potential risk factor for the development of PFOA and the occurrence of AKP is a patellar malalignment (either a translational or a rotational deviation of the patella relative to any axis) which depends on osseus (e.g. patellar tilt (PT), patella alta/baja, trochlea groove etc.) and soft tissue abnormalities (e.g. weakened M. vastus medialis obliquus, tightness of the lateral retinaculum, lesion of the medial retinaculum etc.) [7–10]. Nevertheless, the lack of concordance between patellofemoral malalignment and the development of PFOA shows that, apart from mechanical risk factors, other pathomechanisms must play a role, too [11].

Besides structural characteristics, pathophysiologic processes such as inflamed fat pad tissue, inflamed peripatellar synovial tissue and peripatellar soft tissue neuromas are also described to be involved in the development of patellofemoral pain [12]. Furthermore, Zautra et al. investigated the correlation of acute interpersonal stress levels and depressive symptoms with OA pain [13]. They elucidated that patients with more pronounced depressive symptoms and higher acute stress levels tended to experience more arthritic pain. In this context, the sympathetic nervous system (SNS) and its long-term overactivation in form of chronic stress might play a role. Previous studies have shown that the SNS is involved in OA pathogenesis by differentially affecting bone and cartilage metabolism and remodeling [14]. For example, a predominant catabolic effect of norepinephrine (NE), the major SNS neurotransmitter in the periphery, on articular cartilage has been demonstrated [15–17]. Other studies observed that the activation of SNS lead to subchondral bone loss and the other way around, chemical sympathectomy in mice with surgically induced OA resulted in significantly increased calcified cartilage and subchondral bone plate thickness [18,19]. These studies were mainly performed *in vitro* or in experimental OA models investigating the knee joint or the temporomandibular joint in mice and rats. However, potential correlations between TFOA, PFOA and SNS activity have not yet been analyzed.

Risk factors for the development of knee pain in the context of osteoarthritis remain unclear. Radiological findings often do not correlate with clinical findings, thus, further pathomechanisms in the development and perception of pain must play a role. Therefore, the aim of this study is to investigate the correlation of SNS activity (measured by subjective perception of stress, chronic stress parameters as well as stress related soluble factors) with KOA severity, patellofemoral malalignment, and pain. We hypothesized that radiological OA parameters are associated with higher pain scales, increased scores of chronic stress as well as increased concentrations of stress biomarkers in synovial fluid and serum of OA patients.

## Material and methods

### Study design and setting

The present study describes a cross-sectional study. Forty-seven patients with symptomatic KOA were recruited between 07/2020 and 11/2020 on a voluntary basis. Patients were included if they were planned to receive partial (PKA) or total knee arthroplasty (TKA). After giving their informed consent prior to participation, preoperative patient characteristics were recorded. In addition, all patients were assessed radiologically in three planes prior to operation and detailed radiographic measurements were performed (Table 2). Besides radiological analyses, preoperative knee-specific scoring systems and questionnaires were collected. In order to estimate the preoperative subjective chronic stress level, patients were asked to complete the Perceived Stress Questionnaire (PSQ-20). The radiological measurements, the assessment of functional and subjective scoring systems as well as the surgery were performed at the University Hospital Frankfurt. Preoperatively obtained serum and intraoperatively obtained synovial fluid samples were used to objectively quantify biomarkers of chronic stress. The serum and synovial fluid concentrations of chronic stress markers were determined at the Institute for Clinical Chemistry, University Hospital Cologne. The Ethics Committee of the Johann Wolfgang Goethe University Frankfurt/Main approved the project (vote number 19–347). All investigations were performed in accordance with relevant guidelines and regulations.

### Participants

The study population consisted of 47 caucasian patients with KOA who underwent partial (PKA) or total knee arthroplasty (TKA) between 07/2020 and 11/2020. We excluded patients with rheumatic disease, gout, previous spinal surgery, previous joint infections, previous major surgery of the affected joint or intra-/periarticular tumors.

### Variables and data measurement

#### Radiological measurements

**Fig 1:**
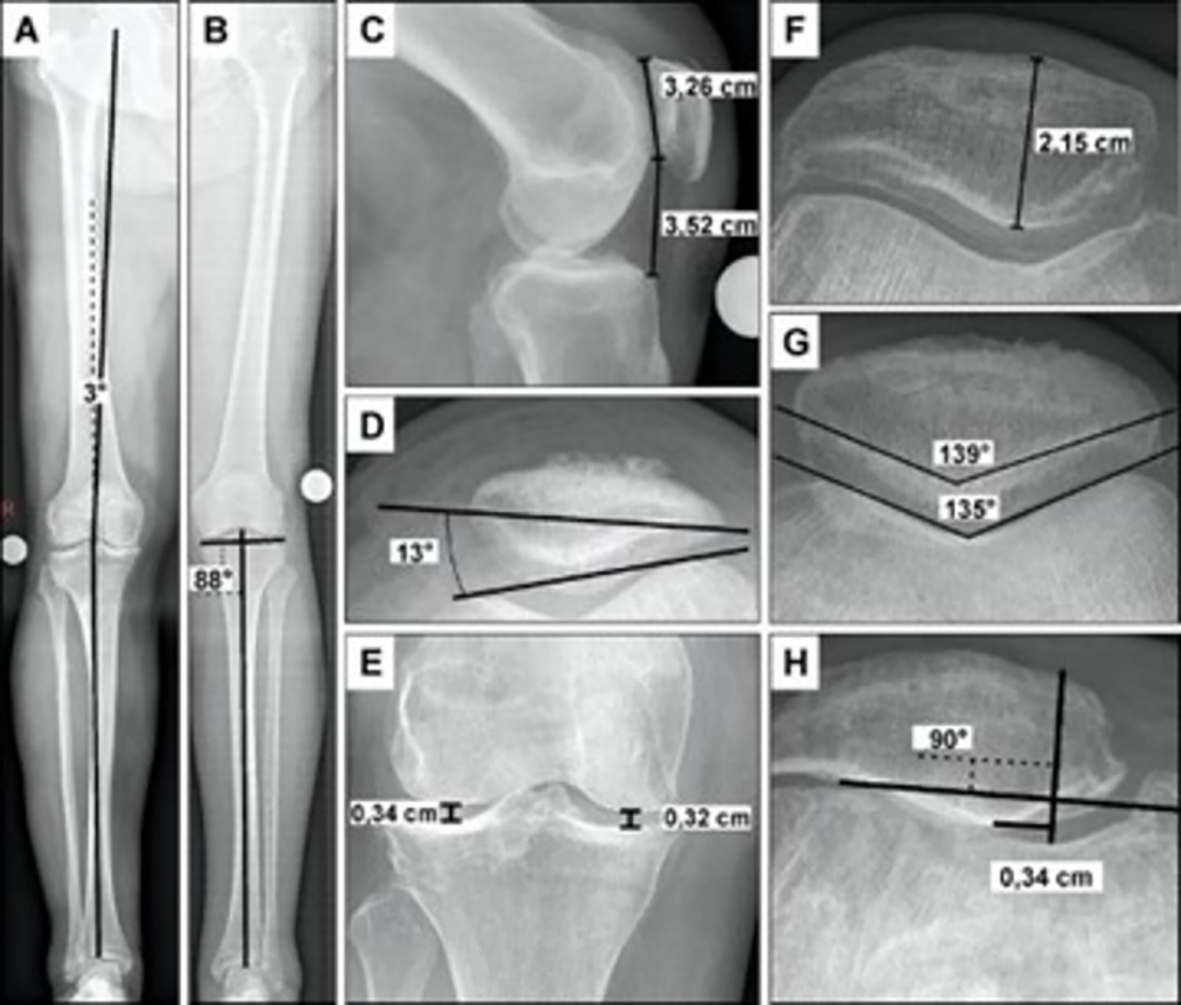
Radiological parameters determined in this study. **(A)** Mechanical femorotibial angle **(B)** Medial proximal tibial angle **(C)** Caton-Deschamps-Index (3,52cm/3,26cm) **(D)** Patellar tilt **(E)** Medial and lateral joint space width **(F)** Patellar thickness **(G)** Sulcus (135°) and facet (139°) angle **(H)** Patellar displacement.

All patients undergoing TKA or PKA were assessed radiologically in three planes (hip-knee-ankle weight bearing anteroposterior, knee lateral, patella supine Merchant view) prior to operation (Fig 1). Weight bearing anteroposterior radiographs were used to determine the lower limb alignment parameters (Mechanical femorotibial angle (MFTA); medial distal femur angle (MDFA); medial proximal tibial angle (MPTA)) as well as the medial and lateral knee joint space. The lateral view was used to measure the Caton-Deschamps-Index (CDI) [20]. Supine Merchant views were performed to assess following patellofemoral measurements: Hepp’s Classification [21], PT, displacement of the patella, patellar thickness, sulcus angle and facet angle. The Kellgren-Lawrence score was used to grade TFOA and PFOA [22,23]. In addition, Merchant classification was used to grade PFOA [24]. The radiographic measurements were performed with a commercially available templating program, TraumaCad® (version 2.3.4.1; Voyant Health, Petach-Tikva, Israel).

#### Knee function, pain and chronic stress level

In addition to radiological analyses, the knee joints of all patients were investigated using a knee-specific scoring system that includes both an objective, physician-derived component and a subjective, patient-derived component (The 2011 Knee Society Knee Scoring System^©^ (KSS^©^)) [25]. Moreover, the Western Ontario and McMaster Universities Osteoarthritis Index^®^ (WOMAC^®^) questionnaire was used to assess pain, stiffness, and functional limitations [26]. To estimate the subjective chronic stress levels of the patients the Perceived Stress Questionnaire (PSQ-20) was collected [27]. Higher values in the subscales of the KSS^©^ and WOMAC^®^ indicate a higher amount of the tested item, respectively. Accordingly, greater perceived stress is expressed by higher PSQ-20 values.

#### Serum and synovial fluid analysis

To evaluate the chronic stress level objectively, biomarkers such as cortisol (CS) [28], aldosterone (ALD) [29] and dehydroepiandrosterone sulfate (DHEA-S) [30] were quantified in serum and synovial fluid samples. In addition, interleukin 6 (IL-6) [31] level was quantified in serum samples. Serum samples were obtained during hospitalization process and synovial fluid was gained during arthroplasty procedure. Synovial fluid sample analysis could be performed only in samples of 14 patients due to absence of joint effusion in the other cases. The serum and synovial fluid concentrations of these markers were determined by competitive ELISA at the Institute for Clinical Chemistry, University Hospital Cologne.

#### Bias

Synovial fluid sample analysis could only be performed in samples of 14 patients due to absence of joint effusion in the other cases. This may have caused attrition bias. Preoperative radiographs for each patient were blinded and templated by an experienced independent observer (MJ) familiar with the templating software.

#### Quantitative variables

**Fig 2:**
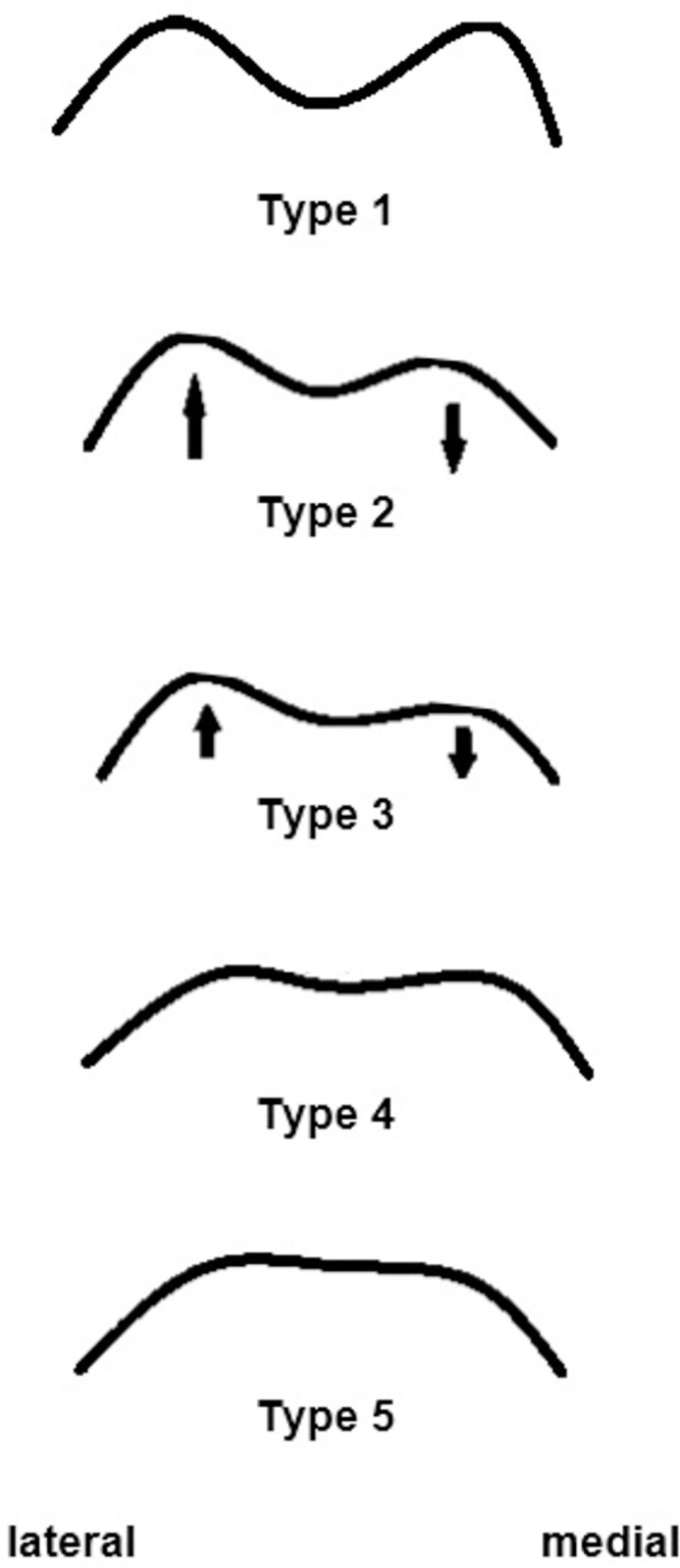
Hepp’s classification: (Type I) Both condyles are approximately the same height and width and almost equally rounded. The sulcus lies approximately in the middle of the trochlea. **(Type II)** The lateral condyle is slightly wider and clearly higher than the medial one. The sulcus depth is shifted somewhat medially. **(Type III)** Hypoplasia of the medial condyle and hyperplasia of the lateral condyle. The sulcus is flattened and clearly shifted medially. Compared to the lateral condyle, the medial one becomes shorter and flatter. **(Type IV)** Flattening of both condyles. The lateral condyle is usually slightly longer and higher than the medial one. The sulcus is flat. **(Type V)** The sulcus is not present. The trochlea is flat and in extreme cases even convex [21].

To investigate whether improper mechanical guidance of the patella is associated with altered tilt values, patients were divided into two groups (≤II and >II) using Hepp’s classification system (Fig 2). Those groups were chosen because the author of the classification system found that 89% of patients without patellar dislocation had type I and II, but 96.5% with patellar dislocation had type III, IV and V [21]. To investigate whether high PT values are associated with higher function and pain scores, patients were divided in two groups (PT <5° and ≥5°) according to Grelsamer et al. [8].

### Statistical analysis

Statistical data analysis was performed using SPSS version 26 (IBM Corporation, New York). The Shapiro-Wilk test was used to test normal distribution of the analyzed parameters. Continuous and normally distributed variables (S1 Table) were presented as means ± standard deviation (SD) and were compared between two groups using the unpaired, Student’s *t*-test (TT). Non parametric variables were presented as medians and interquartile ranges (IQRs) and were compared between two groups using the Mann-Whitney U test (MWU). Correlation analysis was performed by either Pearson-test (r_P_) or Spearman’s rank correlation coefficient (r_S_) depending on normal distribution of the analyzed parameters. All tests were two-sided. Furthermore, we calculated a sensitivity power analysis to evaluate what effect sizes a within-subjects design is sensitive enough to detect. A Pearson’s correlation coefficient and Spearman’s rank correlation coefficient with 47 participants would be sensitive to effects of r = 0.33 with 75% power (alpha = .05, two-tailed). The significance level was set at *p* ≤ 0.05.

## Results

### Patient characteristics and descriptive data

The study population consisted of 47 caucasian patients with KOA who underwent partial (PKA) or total knee arthroplasty (TKA). In total 18 men and 29 women were included. A total of 21 right knee joints and 26 left knee joints were operated. The mean age of the study population was 65.9 years (41–84 years) (Table 1).

**Table 1:** Patient characteristics.

	Mean ± SD
**Age [years]**	66 ± 11
**Height [cm]**	165.2 ± 12.1
**Weight [kg]**	88.2 ± 22.8
**BMI [kg/m^2^]**	31.3 ± 6.9
	**N; %**
**Gender**	
Female	29; 61.7%
Male	18; 38.3%
**Site**	
Right	21; 44.7%
Left	26; 55.3%
**Diabetes**	8; 17.0%
**aHT**	24; 51.1%
**COPD**	1; 2.1%
**Hypothyreosis**	5; 10.6%
**Beta-blocker**	17; 36.2%
**NSAIDs**	6; 12.8%

### Radiographic results

Table 2 shows the radiographic results of the investigated patient study population.

**Table 2:** Radiographic parameters.

Variable	Mean	±SD	Range
MFTA (°) (n = 47)	-1.3	6.2	-14–13
Medial Joint Space TF (mm) (n = 47)	2.6	1.8	0–6
Lateral Joint Space TF (mm) (n = 47)	4.3	1.9	0.5–8.9
Caton-Deschamps-Index (n = 47)	0.9	0.2	0.4–1.3
Medial Joint Space PF (mm) (n = 44)	8.1	2.8	2.6–15.5
Lateral Joint Space PF (mm) (n = 44)	6.0	2.3	0–10.4
PT (°) (n = 47)	6.9	5.7	-7–24
Sulcus Angle (°) (n = 46)	132.1	21.2	0–157
Facette Angle (°) (n = 46)	137.0	7.9	120–155
Patellar displacement (mm) (n = 46)	0.2	0.3	-0.4–1.1
Patellar thickness (mm) (n = 47)	23.9	0.4	1.7–3.4
**Variable**	**Median**	**IQR**	
Tibiofemoral Kellgren-Lawrence score	3	1	1–4
Patellofemoral Kellgren-Lawrence score	2	2	1–4
Patellofemoral Merchant Score	1	1	1–4

**Abbreviations**: MFTA—Mechanical femorotibial angle; TF—Tibiofemoral; PF—Patellofemoral; PT—Patellar tilt; SD—Standard deviation.

### No correlations between TFOA, preoperative pain and chronic stress

To investigate whether TFOA correlates with increased preoperative pain and stress, knee specific questionnaires (KSS^©^ and WOMAC^®^) as well as objective (stress biomarkers) and subjective (PSQ-20) stress levels were examined. No correlation between tibiofemoral Kellgren-Lawrence score and preoperative KSS^©^ Symptoms Score was found (r_S_ = -0.008, p = 0.961). Furthermore, neither stress biomarkers nor subjective stress levels correlated with tibiofemoral Kellgren-Lawrence score or preoperative WOMAC^®^ Score.

### PT correlates with CDI and medial patellofemoral joint space

**Fig 3:**
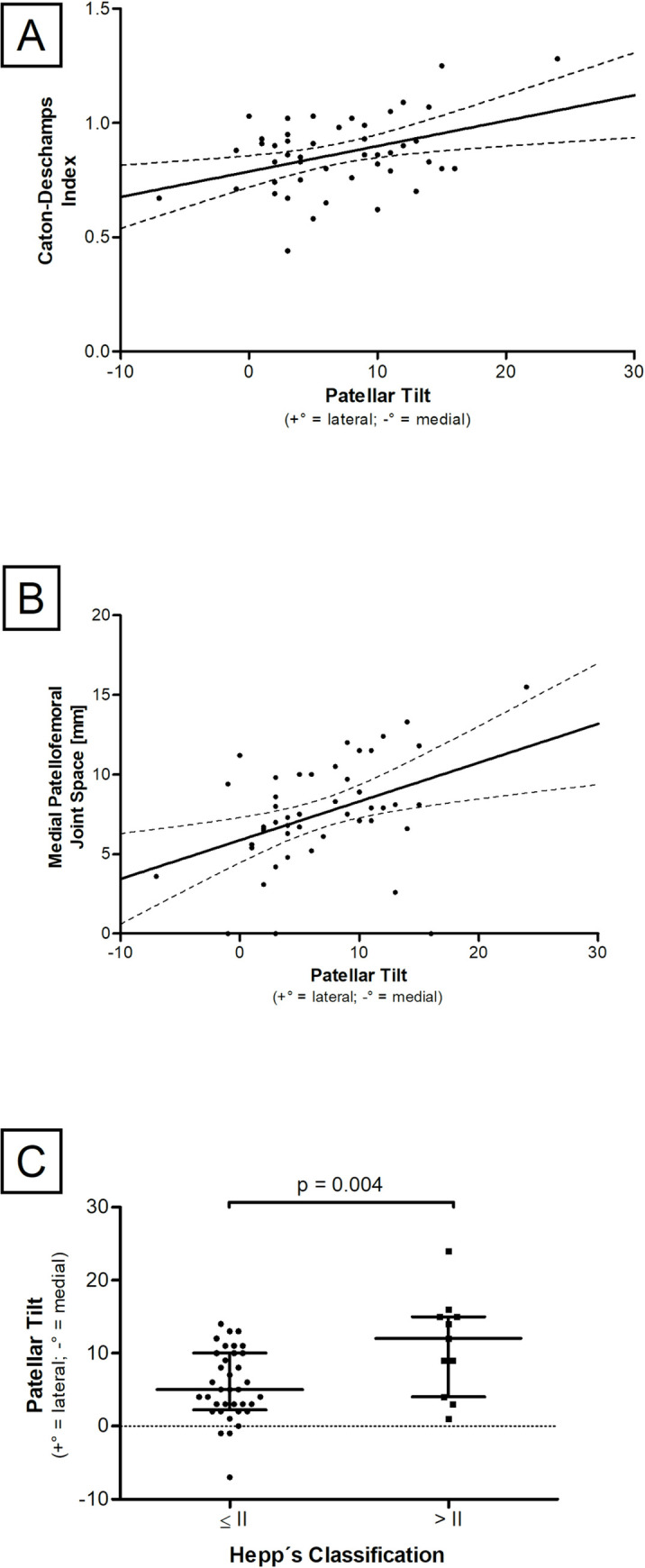
PT and patellofemoral malalignment parameters. **(A)** Scatter diagram of the relationship between PT and CDI. **(B)** Scatter diagram of the relationship between PT and the medial patellofemoral joint space. **(C)** Correlation between both Hepp’s Classification system groups (≤ II and > II) and PT. Each black reference point represents an individual patient. Data represent medians with interquartile range.

Mean PT of the study population was 6.87° (-7° - 24°). PT values correlated significantly with the CDI (r_P_ = 0.394, p = 0.006) (Fig 3A). A high position of the patella (patella alta) correlated with high PT values. In addition, medial patellofemoral joint space was examined (mean 8.1 mm ± 2.8 mm). A significant and positive correlation between PT and medial patellofemoral joint space was found (r_P_ = 0.516, p<0.001)(Fig 3B). To investigate whether improper mechanical guidance of the patella correlates with altered PT values, Hepp’s classification was examined. Our data show a significant relationship of Hepp’s classification and the PT. Patients classified ≤ II (n = 36) showed significant lower PT (MWU: p = 0.014) compared to patients classified > II (n = 11) (Fig 3C). No correlation between PT and coronal alignment of the leg (varus or valgus deformity) was detected (r_P_ = -0.031, p = 0.836).

### Negative correlation of PT and preoperative pain

**Fig 4:**
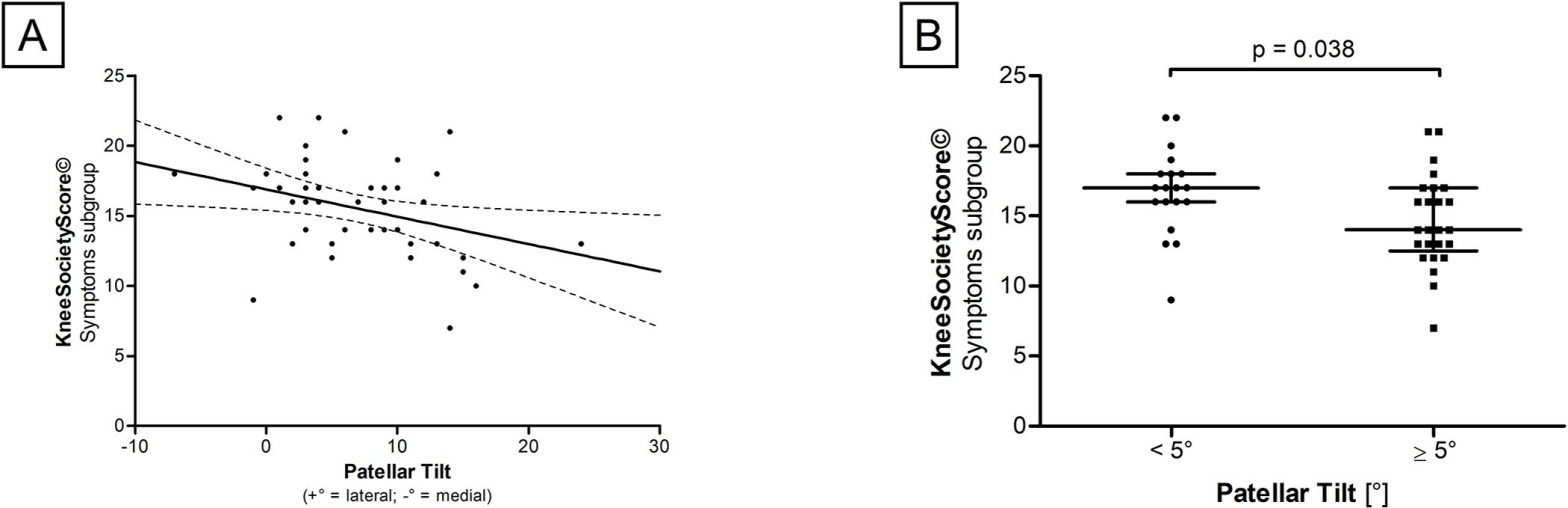
Correlations of PT and pain. **(A)** Scatter diagram of the relationship between PT and KneeSocietyScore©–Symptoms subgroup. **(B)** Correlation between both groups (PT < 5° and PT ≥ 5°) regarding KneeSocietyScore©—Symptoms subgroup score. Each black reference point represents an individual patient. Data represent medians with interquartile range.

To investigate whether high PT values correlate with altered preoperative pain levels, KSS^©^ and WOMAC^®^ scores were examined. A significant negative correlation of PT with KSS^©^—symptoms subgroup (r_P_ = -0.340, p = 0.024) was detected (Fig 4A). Furthermore, a significant difference in KSS^©^—Symptoms subgroup between patients with a PT < 5° (n = 19) and a PT ≥ 5° (n = 25) was found. Patients with PT < 5° had significantly higher scores in the KSS^©^–symptoms subgroup (TT: p = 0.038) (Fig 4B). No correlations between PT and WOMAC^®^ scores were found. Table 3 shows the results of KSS^©^, WOMAC^®^ and PSQ-20 questionnaires.

**Table 3:** Results of questionnaires.

Variable	Mean	±SD	Range
KSS^©^ Expectations (n = 44)	13.70	1.9	6–15
KSS^©^ Satisfaction (n = 45)	11.64	5.26	0–26
KSS^©^ Symptoms (n = 44)	15.55	3.35	7–22
KSS^©^ Function (n = 44)	37.80	18.86	1–77
WOMAC^®^ Pain (n = 47)	30.47	7.67	13–42
WOMAC^®^ Stiffness (n = 47)	13.34	4.26	4–20
WOMAC^®^ Activity (n = 46)	100.89	31.28	33–157
PSQ-20 (n = 47)	41.72	13.28	18–75

**Abbreviations:** KSS–Knee Society Score^©^; WOMAC^®^—Western Ontario and McMaster Universities Osteoarthritis Index^®^; PSQ—Perceived Stress Questionnaire; SD—Standard deviation.

### Correlations of PFOA parameters and chronic stress biomarkers

**Fig 5:**
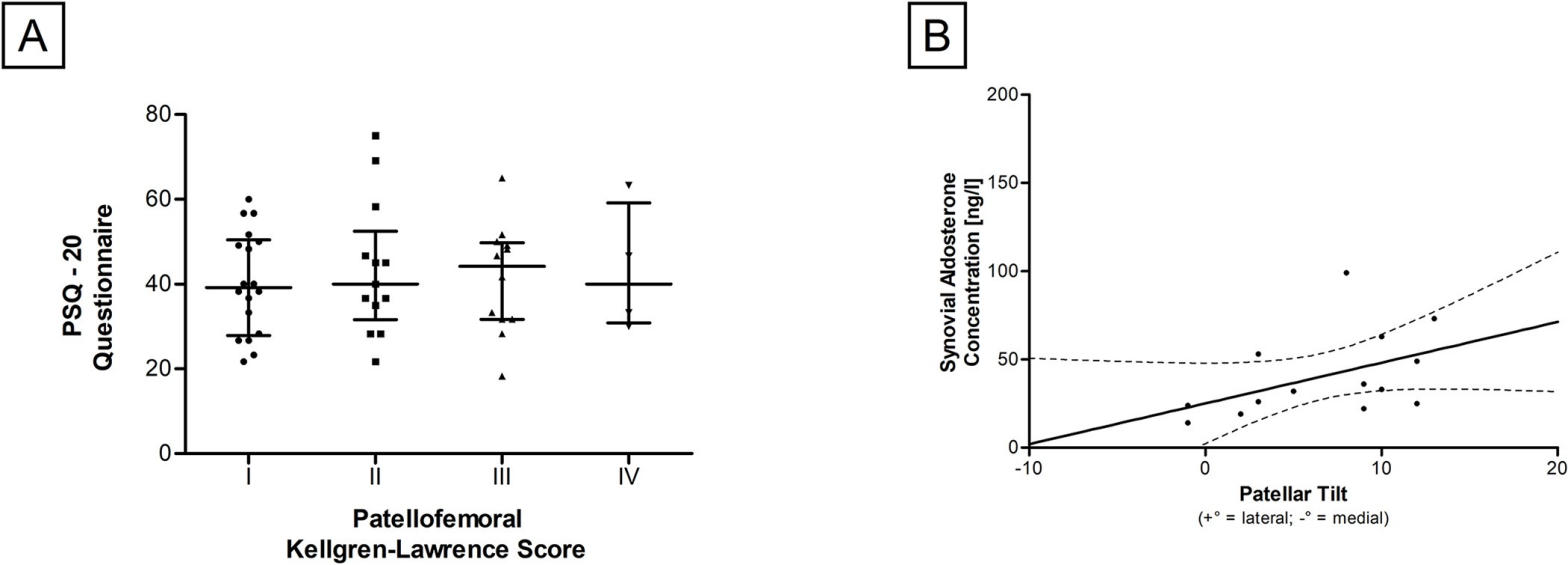
Correlations of PFOA parameters and chronic stress parameters. **(A)** Relationship of Kellgren-Lawrence score and PSQ-20 questionnaire results. Patients were divided in 4 groups according to patellofemoral Kellgren-Lawrence score **(B)** Scatter diagram of the relationship between PT and synovial ALD concentration. Each black reference point represents an individual patient. Data represent medians with interquartile range.

To investigate whether PFOA and PT correlate with increased chronic stress levels, objective (stress biomarkers) and subjective (PSQ-20) stress levels were examined. No correlations between PFOA and PSQ results (patellofemoral Kellgren.Lawrence Score: r_S_ = 0.045, p = 0.763; patellofemoral Merchant-Score r_S_ = 0.144, p = 0.333) (Fig 5A) or chronic stress biomarkers in serum samples (ALD / IL-6 / CS) were detected. In contrast, a positive and significant correlation between ALD levels in synovial fluid and the PT was observed (r_S_ = 0.548, p = 0.042) (Fig 5B). Table 4 summarizes the results of chronic stress biomarker analysis.

**Table 4:** Stress biomarkers.

Biomarker	Median	25% percentile	75% percentile
ALD Serum (ng/l) (n = 40)	85	67	121
ALD Synovial fluid (ng/l) (n = 14)	33	24	53
IL-6 Serum (ng/l) (n = 9)	4	2	6
IL-6 Synovial fluid (ng/l) (n = 15)	300	98	817
CS Serum (μg/l) (n = 41)	116	97	145
CS Synovial fluid (μg/l) (n = 16)	41	19	60
DHEA-S Serum (mg/l) (n = 41)	0.8	0.6	1.5
DHEA-S Synovial fluid (mg/l) (n = 16)	0.4	0.25	0.8

**Abbreviations:** ALD–Aldosterone; IL—Interleukin; CS—Cortisol; DHEA-S—Dehydroepiandrosterone sulfate; SD—Standard deviation.

## Discussion

The SNS is part of the autonomic nervous system and its main function is to stimulate the body’s fight-or-flight response [32]. Its long-term overactivation in form of chronic stress might play a role in OA pathogenesis since several studies revealed that the SNS is involved in bone and cartilage metabolism and that its activation leads to loss of subchondral bone as well as induces a catabolic phenotype in chondrocytes *in vitro* [14,17,19]. In addition, there is evidence that the SNS is involved in osteoarthritic nociception [33].

Therefore, we investigated whether an increased TFOA Kellgren-Lawrence grade correlates with increased sores of chronic stress and an increased release of stress biomarkers in patients’ synovial fluids and sera. Our data does not show any correlations between radiographic TFOA Kellgren-Lawrence grade, serum biomarkers as well as stress questionnaires. In contrast, a prior study demonstrated correlations between radiographic OA parameters, pain questionnaire scores (visual analogue scale) and biomarkers of cartilage degeneration (urinary C-telopeptide fragments of type II collagen) [34]. One explanation for this could be the relatively small number of synovial samples examined (n = 14). For this reason, that part of our hypothesis has to be rejected. Nevertheless, it is attractive to speculate that the SNS, activated by chronic stress, does not only affect the tibiofemoral but also the patellofemoral joint. Literature shows that radiological consideration only of the tibiofemoral compartment can lead to neglect of up to 24% of patients with knee pain [35]. Therefore, and because of discarding the primary hypothesis, detailed analyses of the patellofemoral compartment were performed.

An aberrant dispersion of patellofemoral joint reaction force caused by patellar malalignment is described as a potential risk factor for the development of PFOA and the occurrence of AKP [7,8]. Therefore, we investigated correlations between malalignment parameters such as PT and CDI with subjective perception of pain and stress, chronic stress parameters and stress related soluble factors. In a first step, we found a positive correlation between patella alta (high CDI values) and PT. Ward et al. confirmed our results and described that patients with patella alta had more lateral PT and less contact area than subjects with normal patella position under weightbearing conditions [36]. Patella alta is thought to be an important malalignment parameter and predispose individuals to patellofemoral pain [11,36,37]. It is assumed that a high position of the patella engages the trochlear groove at a greater knee flexion angle [38]. This may lead to a lateral malalignment and less medial-lateral restriction of the patella at low knee flexion angles. Subsequently, lateral malalignment decreases patellofemoral contact area and finally results in an increased joint stress and pain [39,40]. In this context, Pal et al. demonstrated a positive correlation between pain, patellar height and patellar malalignment [11]. In contrast to the findings of Pal et al., we did not detect a positive correlation between high PT values and high scores in the KSS^©^ symptoms subgroup. However, we were able to elucidate that PT <5° is associated with higher scores in the KSS^©^ symptoms subgroup. According to Grelsamer et al., normal PT values are considered to be lower 5° [8]. Other studies declare PT values of 8–15° as normal [41]. In summary, there is no consensus on the physiological PT values and our findings suggest that PT values of <5° might be a risk factor for stronger symptoms in patients with PFOA.

Furthermore, our data revealed that improper mechanical guidance of the patella (Hepp’s classification > II) is associated with significant higher PT values and a higher medial patellofemoral joint space. Conversely, this means that strong medial-lateral restriction of the patella due to deep femoral trochlear groove (Hepp’s classification < II) is associated with lower PT values and a narrow medial patellofemoral joint space. Subsequently, a simultaneously ongoing degeneration of the patellofemoral joint may further decrease the medial joint space and finally result in an increased mechanical stress of the joint and pain. In this context, Ijima et al. found that PT was lower in patients with PFOA and TFOA compared to patients with TFOA only [42]. Therefore, even low PT values might contribute to the pain levels of patients with TFOA and have to be considered. In this context, it is important to mention that all patients in this study were scheduled for a TKA. These patients are considered to be at the end of the symptom severity range since they are about to undergo a major surgery. Therefore, from our study no conclusions can be drawn about patients with less severe symptoms without indicated surgery. Previous studies revealed that in spite of modern implants and advanced surgical techniques the dissatisfaction rate after total knee arthroplasty remains at 20% [43]. Nakano et al. gave different explanations for this fact one being the management of the patella. In this context it is known that correct management of the PT plays a role in preventing complications [43]. Thus, detailed knowledge of PT and its pathophysiology is important to prevent pain and postoperative complications. The results of the present study may indicate that even low PT values play a role in pain perception and postoperative dissatisfaction. Future studies should test this hypothesis prospectively in postoperative patients.

Furthermore, our data showed a positive correlation between synovial ALD levels and PT. Since we found no correlation between tibiofemoral pathologies and serum or synovial fluid chronic stress biomarkers, our findings may indicate that PT correlates with increased synovial fluid ALD levels. It is interesting to speculate, that a long-term overactivation of the SNS in form of chronic stress (high ALD levels) might have an influence on the tension of the knee extensor muscles [44]. This could lead to an increased PT and an aberrant dispersion of patellofemoral joint reaction forces. The hypothesis that SNS exerts its effects not only via influencing bone and cartilage metabolism but also indirectly via muscle activation would need to be tested in future studies. Nevertheless, since the knee consists of two joints, it is generally difficult to distinguish which degeneration is mainly responsible for the release of chronic stress biomarkers and future studies need to address this problem in a larger study population.

There are a few limitations of this study. One limitation is the absence of a control group. For ethical reasons, it is not justifiable to aspirate an undegenerated and healthy human knee by taking a sample for study reasons. Since no healthy human knee aspirates were available, it was not possible to compare healthy with KOA stages. In addition, synovial fluid sample analysis could be performed only in samples of 14 patients due to absence of joint effusion in the other cases. Therefore, there might be a lack of power to draw definitive conclusions from the synovial analysis results. Nevertheless, a possible correlation between PT and synovial ALD level is an interesting finding that should be proven and further investigated in future studies. Given the large number of comparisons in this study, some findings could occur by chance. Therefore, this study should be considered an exploratory analysis with the goal of initiating follow-up studies with larger patient populations.

## Conclusions

We show that lower PT values are accompanied with higher scores in the symptom subgroup of the KSS^©^. As this is against the initial hypothesis, the present pilot study may indicate that the previous specification of standard values must be reconsidered, as even low PT values seem to play a role in occurrence of PFOA symptoms. Further prospective studies with a larger and broader patient population (including patients with mild and moderate symptoms) are needed to add value to the current inconsistent literature. We demonstrated increased synovial fluid ALD levels in patients with high PT and identified possible relations between SNS and PFOA. For the verification of the influences of SNS on PFOA and TFOA experimental approaches might me more favorable. The results will contribute to a better understanding of cellular processes of OA that might lead to the identification of novel targets for therapeutic approaches.

## Supporting information

S1 Table Test of normality. (DOCX)

S2 Table Correlation table for study parameters. (DOCX)

S1 File Minimal underlying data set. (XLSX)
