# Supplementary material for: Patellar malalignment correlates with increased pain and increased synovial stress hormone levels–A cross-sectional study
Source: PLoS One. 2023 Jul 27;18(7):e0289298. doi: 10.1371/journal.pone.0289298 (PMC10374142; doi:10.1371/journal.pone.0289298)
Supplement: S1 Table — (DOCX) [file pone.0289298.s001.docx]

|  | **N** | **Mean** | **SD** | **Median** | **Interquartile Range** | **Shapiro-Wilk** |
| --- | --- | --- | --- | --- | --- | --- |
| **Tibiofemoral Kellgren-Lawrence score** | 47 | 2,79 | 0,657 | 3 | 1 | p < 0,001 |
| **Patellofemoral Kellgren-Lawrence score** | 47 | 2,04 | 0,999 | 2 | 2 | p < 0,001 |
| **Patellofemoral Merchant Score** | 47 | 1,47 | 0,776 | 1 | 1 | p < 0,001 |
| **KSS^©^ Symptoms** | 44 | 15,55 | 3,351 | 16 | 5 | p = 0,475 |
| **WOMAC^®^ Pain** | 47 | 30,47 | 7,672 | 30 | 12 | p = 0,087 |
| **PSQ-20** | 47 | 41,72 | 13,28 | 40 | 18,33 | p = 0,413 |
| **ALD Serum (ng/l)** | 40 | 93,28 | 40,21 | 84,5 | 55 | p = 0,141 |
| **ALD Synovial fluid (ng/l)** | 14 | 40,57 | 24,17 | 32,5 | 32 | p = 0,052 |
| **IL-6 Serum (ng/l)** | 9 | 5,22 | 4,236 | 4 | 6 | p = 0,022 |
| **IL-6 Synovial fluid (ng/l)** | 15 | 442,2 | 391,81 | 300 | 719 | p = 0,028 |
| **CS Serum (µg/l)** | 41 | 118,8 | 50,131 | 116 | 56 | p = 0,026 |
| **CS Synovial fluid (µg/l)** | 16 | 42,69 | 27,913 | 41 | 43 | p = 0,438 |
| **DHEA-S Serum (mg/l)** | 41 | 1,13 | 0,83 | 0,8 | 1,12 | p = 0,002 |
| **PT (°)** | 47 | 6,87 | 5,705 | 6 | 8 | p = 0,233 |
| **Caton-Deschamps-Index** | 47 | 0,864 | 0,161 | 0,86 | 0,19 | p = 0,749 |
| **Medial Joint Space PF (mm)** | 44 | 8,061 | 2,775 | 7,7 | 3,4 | p = 0,656 |
| **MFTA (°)** | 47 | -1,28 | 6,234 | -2 | 8 | p = 0,301 |
| **Hepp Classification** | 47 | 1,81 | 0,97 | 2 | 1 | p < 0,001 |

**S1 Table** Test of normality
