# Supplementary material for: Patellar malalignment correlates with increased pain and increased synovial stress hormone levels–A cross-sectional study
Source: PLoS One. 2023 Jul 27;18(7):e0289298. doi: 10.1371/journal.pone.0289298 (PMC10374142; doi:10.1371/journal.pone.0289298)
Supplement: S2 Table — (DOCX) [file pone.0289298.s002.docx]

**S2 Table** Correlation table for study parameters

|  | CDI | medial PF joint space | MFTA | KSS© - symptoms subgroup | WOMAC® - pain | PSQ | ALD | IL-6 | CS |
| --- | --- | --- | --- | --- | --- | --- | --- | --- | --- |
| PT | r_P_=0.394 (p=0.006) | r_P_=0.516 (p<0.001) | r_P_=-0.031 (p=0.836) | r_P_=-0.340 (p=0.024) | r_P_=-0.005 (p=0.0976) |  | r_P_=0.049 (p=0.765) | r_P_=0.330 (p=0.358) | r_P_=-0.210 (p=0.187) |
| PF KL Score |  |  |  | r_S_=-0.221 (p=0.150) | r_S_=-0.089 (p=0.552) | r_S_=0.045 (p=0.763) | r_S_=0.014 (p=0.933) | r_S_=-0.091 (p=0.458) | r_S_=0.252 (p=0.112) |
| PF Merchant Score |  |  |  | r_S_=-0.163 (p=0.292) | r_S_=-0.170 (p=0.254) | r_S_=0.144 (p=0.333) | r_S_=0.073 (p=0.656) | r_S_=-0.541 (p=0.133) | r_S_=0.006 (p=0.972) |
| TF KL Score |  |  |  | r_S_=-0.008 (p=0.961) |  |  |  |  |  |

PT – Patellar tilt; PF – Patellofemoral; TF – Tibiofemoral; KL - Kellgren-Lawrence; CDI - Caton-Deschamps-Index; MFTA - Mechanical femorotibial angle; ALD – Aldosterone; IL - Interleukin; CS – Cortisol; PSQ – Perceived stress questionnaire.
